# The clinical features and outcomes of diabetes patients infected with COVID-19: a systematic review and meta-analysis comprising 192,693 patients

**DOI:** 10.3389/fmed.2025.1523139

**Published:** 2025-01-29

**Authors:** Kai Liu, Shu Liu, Ting-ting Xu, Hong Qiao

**Affiliations:** ^1^Department of Endocrinology and Metabolism, The Second Affifiliated Hospital of Harbin Medical University, Harbin, China; ^2^Physical Examination Center, The Fourth Affifiliated Hospital of Harbin Medical University, Harbin, China; ^3^Health Management Centre, Fourth Affiliated Hospital of Harbin Medical University, Harbin, Heilongjiang, China

**Keywords:** COVID-19, SARS-CoV-2, diabetes, mortality, clinical features, meta-analysis

## Abstract

**Objectives:**

We sought to explore the relevance of analyses that include critical laboratory parameters and drug treatment, clinical characteristics of diabetic patients who are infected with COVID-19, to the development of individualized treatment strategies for diabetic patients infected with COVID-19.

**Methods:**

We searched Cochrane, Embase, FMRS, Pubmed, Springer, Web of Science databases for systematic reviews and meta-analyses to estimate the clinical characteristics and prognosis of confirmed covid-19 infections in patients with and without diabetes.

**Results:**

Our meta-analysis included a total of 32 studies with 192,693 COVID-19 patients. Common comorbidities in the diabetic group were hypertension, cerebrovascular disease, chronic kidney disease and cardiovascular disease. We discovered that white blood cell count, neutrophil count, inflammatory marker levels, D-dimer, urea, precursor of the brain natriuretic peptide (Pro-BNP) increased and lymphocyte count, estimated glomerular filtration rate (eGFR), albumin decreased significantly in the diabetic group in laboratory test results. Compared with the non-diabetic group, the diabetic group had a higher incidence of complications in acute respiratory distress syndrome (ARDS), shock, acute heart injury, acute kidney injury and more regularly used oxygen therapy, invasive ventilation, non-invasive ventilation, continuous renal replacement therapy (CRRT), extracorporeal membrane oxygenation (ECMO) treatment. Mortality and intensive care unit (ICU) hospitalization rates were highest in the diabetic group than in the non-diabetic group (*p* < 0.05).

**Conclusion:**

Diabetic patients hospitalized with COVID-19 have an increased risk of death, lower discharge rates, and higher ICU admission rates. Their presence of hypertension, cerebrovascular disease, chronic kidney disease (CKD), higher levels of inflammatory markers. Multiple complications are all predictors of poor outcomes in people with diabetes. Our findings will help identify elevated risk factors in diabetics, which will benefit early prediction.

## Introduction

The COVID-19 caused by severe acute respiratory syndrome coronavirus 2 (SARS-CoV-2) infection, it is a novel and serious global health threat that is spreading rapidly across the globe, with confirmed infections and death. The number of infections is growing, with around 500 million confirmed cases worldwide and more than 6 million deaths. Most patients have mild symptoms, but some may develop severe complications, including ARDS, multiple organ failure, septic shock and hypercoagulability, which may eventually result in death (1–3). While large-scale vaccine production has provided a glimmer of hope for humanity for now, the absence of global vaccination and the continued mutation of the virus make eradication of SARS-CoV-2 challenging. Among them, the largest COVID-19 study in the United States found that among 5,700 hospitalized patients with COVID-19, diabetes was one of the most common comorbidities (33.8%), and chronic disease comorbidities had a significant impact on the clinical outcomes of patients with COVID-19 (4). Studies have shown that people with underlying comorbidities of diabetes are more likely to experience adverse outcomes from COVID-19. The COVID-19 pandemic has placed a huge burden on healthcare facilities, especially for the patients who are with them. Most studies report that diabetes is associated with a higher risk of serious events and mortality (5, 6), while others have no clear association (7, 8), so whether diabetes is associated with adverse outcomes in COVID-19 patients controversy remains. This inconsistency may be related to different sample sizes, different populations, and varying levels of confounding adjustment. Numerous articles show the clinical features of COVID-19 patients in various countries (9, 10), but few studies specifically compare the clinical features of COVID-19 in diabetic and non-diabetic patients. This study can provide information on risk factors by correlative analysis of data on essential laboratory parameters and drug treatment for COVID-19 patients with and without diabetes, while helping inform the development of tailored treatment strategies for diabetic COVID-19 patients.

## Methods

### Literature search: identification and selection of studies

The protocol for this systematic review and meta-analysis is available online at PROSPERO; registration number CRD42022312394.

**Figure 1:**
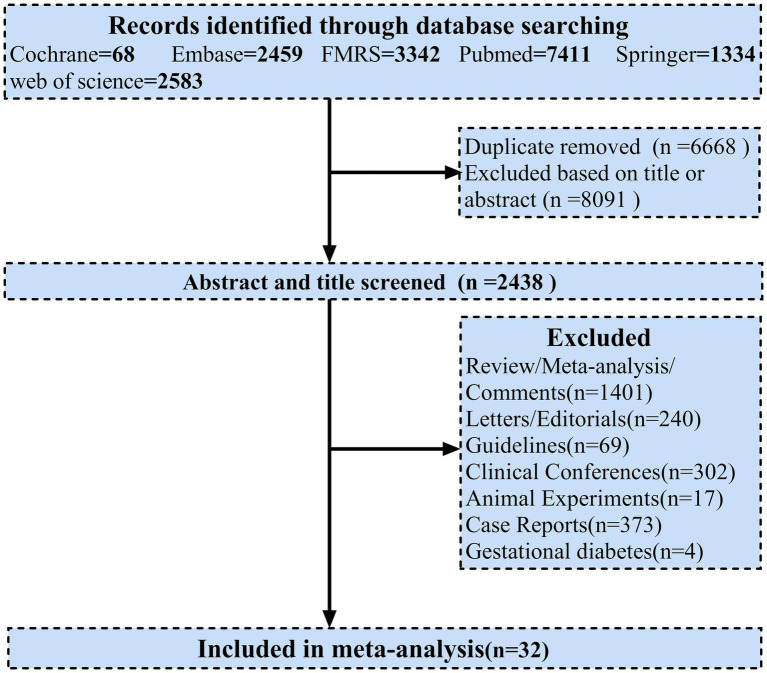
Flow diagrams for literature selection.

All procedures utilized in systematic review and meta-analysis were in accordance with the Preferred Reporting Items for Systematic Reviews and Meta-Analyses (PRISMA) guidelines. A comprehensive search was performed on Cochrane, Embase, FMRS, Pubmed, Springer, Web of Science databases between December 1, 2019 and April 1, 2022. Titles and abstracts of potentially eligible articles were manually reviewed and potentially relevant articles were assessed for eligibility. Two investigators (KL and SL) independently searched for studies. In the event of disagreement over study eligibility, a third investigator (HQ) was required to participate in order to reach consensus. Related unpublished clinical trial results were similarly manually searched for additional potential studies. We searched using a combination of the following keywords: “COVID-19,” “SARS-CoV-2,” “coronavirus,” “2019-nCoV,” “diabet*,” “T1DM” and “T2DM.” The PRISMA flowchart was used to present the search strategy and studies included in the meta-analysis (Figure 1). PRISMA 2020 (Supplementary Table 4), Meta-analysis of Observational Studies in Epidemiology (MOOSE) (Supplementary Table 5) were also adhered to for reporting.

### Inclusion and exclusion criteria

Among the patients included were a significant number of people with type 2 diabetes, most of whom had previously been diagnosed with diabetes, and the remainder who were newly diagnosed with diabetes on admission. For studies to be included, the following inclusion criteria were applied: (a) age ≥ 18 years; (b) cohort studies reporting clinical characteristics of patients with confirmed SARS-CoV2 infection in both diabetic mellitus (DM) and non-diabetic mellitus (non-DM) groups or case–control studies; (c) analysis of one or more clinical characteristics, including demographic characteristics, clinical symptoms, laboratory findings, comorbidities, treatments, outcomes of complications; (d) confirmed patients in a hospital setting, and (f) studies with excellent methodological design (appropriate sample size is considered to be at least more than 20 patients per group). In addition, the following criteria were used to exclude studies: (a) non-human/animal studies; (b) duplicate publications; (c) no full text articles; (d) case reports, guidelines, clinical meetings, letters, systematic reviews and meta-analysis; (e) studies that did not provide diabetes and non-diabetic related data or related clinical outcomes.

### Data extraction

Two researchers (KL and SL) independently extracted data from eligible studies to minimize bias. Any disagreements will be discussed with a third investigator (HQ) to reach consensus. We extracted and analyzed items from eligible studies, including country, year, publication date, number of reported cases, sex, age, clinical signs and symptoms, comorbidities, laboratory findings, complications and outcomes of infected patients.

### Quality assessment of including studies

All articles were independently evaluated and compared by two raters. Any inconsistencies should be considered or further consulted by an independent expert. We used the Newcastle-Ottawa Scale (NOS) to assess the risk of bias of included studies (Supplementary Table 1), and a NOS score greater than 7 was considered to be of decent quality (11).

### Statistical analysis

After STATA 17.0 software analyses, the Odds ratio (OR) and the corresponding 95% confidence interval (CI) of the relevant factors in each study are calculated. Heterogeneity between studies was assessed using Cochrane Q and I^2^ statistics. I^2^ reflects the fraction of heterogeneity in the total variation of the effect sizes. Values <25% indicate low heterogeneity, values between 25 and 50% indicate moderate, > 50% strong heterogeneity. If I^2^ is greater than 50%, indicating greater heterogeneity, the pooled SMD values and the corresponding 95% CI are calculated using the DerSimonian-Laird method using a random effects model. If I^2^ is less than 50 percent, the fixed effect model is calculated. The Egger’s test is used to assess publication bias, which is suspected if the Egger’s test have a *p*-value <0.05. The sensitivity analysis was performed after a stepwise exclusion of studies, followed by a comparison of the raw results with those from the re-analysis to confirm the stability of our primary meta-analysis. If the combined effect point falls within the confidence interval of the total effect size, the analysis results are robust and reliable, we need to be careful in interpreting the results and drawing conclusions if the combined effect point falls outside the confidence interval for the total effect size, or if the combined effect point differs significantly from the total effect size.

## Results

### Literature search and characteristics of including studies

In order to identify these possible risk factors and severity predictors that could be useful for clinical treatment in the future treatment of patients with diabetes affected by COVID-19, we used a meta-analysis that combined demographic and clinical characteristics from each study. Some outcomes including gender, age, symptoms, complications, comorbidities, treatment, laboratory measures and clinical outcomes were observed to differ between DM and non-DM cases. A total of 17,197 records were identified from the database. After excluding duplicates, the titles and abstracts of 2,438 articles were screened, we from the articles screened from literature and online sources, a total of 32 articles were included after exclusion (31 retrospective studies and 1 prospective study) eligible for inclusion prespecified criteria for analysis (Figure 1). Numbers ranged from 29 (the smallest study) to 33,478 (the largest study). Overall, our systematic review included 192,693 individuals. Most studies were conducted in Asia (China, *n* = 14; South Korea, *n* = 3; Iran, *n* = 3; Kuwait, n = 1; Saudi Arabia, *n* = 1; United Arab Emirates, *n* = 1), while in North America (United States, *n* = 2) and 6 studies in Europe (France, *n* = 2; Italy, *n* = 1; Denmark, *n* = 1; turkey, *n* = 2) (Table 1). All articles included in the meta-analysis were of high quality according to the NOS tool, as described in Supplementary Table 3.

**Table 1:** Basic information of included studies.

Study	Year	Country	Study design	Total patients (non-DM/DM)	Overall age	non-DM Age (mean ± SD)	DM age (mean ± SD)	Sex (Male/Female)	Literature quality
Shi et al. (1)	2020	China	Retrospective	306 (153/153)	64 ± 11.9	64 ± 11.9	64 ± 11.9	156/150	9
Akbariqomi et al. (2)	2020	Iran	Retrospective	595 (447/148)	56.3 ± 16	57.4 ± 16.3	53.2 ± 14.9	401/194	9
Khalili et al. (3)	2020	Iran	Retrospective	254 (127/127)	65.7 ± 12.5	65 ± 12.5	66.4 ± 12.5	142/112	8
Demirci et al. (4)	2021	Turkey	Retrospective	148,586 (115,108/33478)	41.6 ± 32.2	38 ± 15.5	54 ± 60	77,912/70674	8
Alshukry et al. (6)	2021	Kuwait	Retrospective	417 (273/144)	45.3 ± 17	39.55 ± 16.59	56.44 ± 11.64	262/155	7
Calvisi et al. (27)	2021	Italy	Prospective	169 (118/51)	63.2 ± 19.1	63 ± 20.6	70 ± 13.0	113/56	7
Cheng et al. (28)	2020	China	Retrospective	236 (133/103)	54.5 ± 19.2	48 ± 20.9	63 ± 12.7	128/108	7
Yan et al. (29)	2020	China	Retrospective	193 (145/48)	61.9 ± 18.5	57 ± 20.9	69 ± 11.4	114/79	8
Zhang et al. (30)	2020	China	Retrospective	145 (84/61)	62 ± 14.5	59.4 ± 16.0	65.6 ± 11.4	74/71	9
Kim SW et al. (31)	2021	Korea	Retrospective	1,019 (802/217)	59 ± 17.5	56.4 ± 18.0	68.7 ± 11.2	352/667	7
Cai et al. (32)	2020	China	Retrospective	941 (818/123)	57.4 ± 56.7	56.3 ± 57.1	64.7 ± 54	454/487	8
Chen et al. (33)	2020	China	Retrospective	563 (476/87)	51.5 ± 20.5	49.2 ± 20.8	64.2 ± 12.8	NA	9
Vasbinder et al. (34)	2022	United States	Retrospective	2044 (1,358/686)	60 ± 16.3	58 ± 17	64 ± 14	1191/853	7
Elemam et al. (35)	2021	United Arab Emirates	Retrospective	350 (239/111)	47.4 ± 14.4	44.6 ± 14.3	53.7 ± 12.7	274/76	8
Cheng et al. (36)	2021	China	Retrospective	407 (357/50)	47.3 ± 16.3	46.2 ± 16.3	55.2 ± 14.0	195/212	8
Zhang et al. (37)	2020	China	Retrospective	250 (166/84)	52.8 ± 20.5	48.1 ± 22.4	62.3 ± 11.3	106/144	8
Ling et al. (38)	2020	China	Retrospective	702 (651/51)	42.4 ± 15.5	41.2 ± 15.1	58.4 ± 11.2	384/318	7
Kim MK et al. (39)	2020	Korea	Retrospective	1,082 (847/235)	59 ± 17.5	56.5 ± 18.0	68.3 ± 11.9	384/698	8
Han et al. (40)	2020	China	Retrospective	306 (177/129)	59.2 ± 16.4	55.0 ± 17.9	65 ± 11.9	174/132	7
Li et al. (41)	2020	China	Retrospective	199 (123/76)	62 ± 15.5	57.9 ± 15.7	68.7 ± 12.8	110/89	8
You et al. (42)	2020	Korea	Retrospective	5,473 (4,978/495)	NA	NA	NA	2439/3034	9
Sun et al. (43)	2020	China	Retrospective	1,618 (1,392/226)	55.2 ± 15.7	54.2 ± 16.3	61.5 ± 9.6	733/885	7
Yang et al. (44)	2021	China	Retrospective	1,247 (572/675)	61.2 ± 14.3	64.2 ± 12.6	58.7 ± 15.2	598/649	7
Chen et al. (45)	2020	China	Retrospective	208 (112/96)	62.8 ± 11	61.3 ± 12	64.6 ± 9.7	101/107	9
Sutter et al. (46)	2021	France	Retrospective	1,206 (603/603)	71.1 ± 14.5	71.3 ± 15.9	71 ± 13	745/461	8
Alguwaihes et al. (26)	2020	Saudi Arabia	Prospective	439 (139/300)	NA	NA	NA	2439/3034	7
Bode et al. (47)	2020	United States	Retrospective	1,122 (671/451)	60.3 ± 58.2	59.9 ± 61.6	61.1 ± 52.8	624/498	7
Al-Salameh et al. (48)	2021	France	Retrospective	432 (317/115)	72.1 ± 17.6	71.9 ± 18.6	72.8 ± 14.6	238/194	8
Mansour et al. (49)	2020	Iran	Retrospective	353 (242/111)	61.6 ± 16.3	60.7 ± 17.5	63.6 ± 13.3	203/150	8
Chung et al. (50)	2020	Korea	Retrospective	110 (81/29)	56.8 ± 16.9	53.5 ± 17.9	66.3 ± 8.9	48/62	9
Alhakak et al. (51)	2022	Denmark	Retrospective	3,295 (2,117/1178)	72.3 ± 15.2	71.9 ± 16.8	73.2 ± 11.8	1853/1442	8
Sonmez et al. (52)	2021	Turkey	Retrospective	18,426 (9,213/9213)	NA	NA	NA	7980/10446	9

### Demographic and clinical characteristics

**Figure 2:**
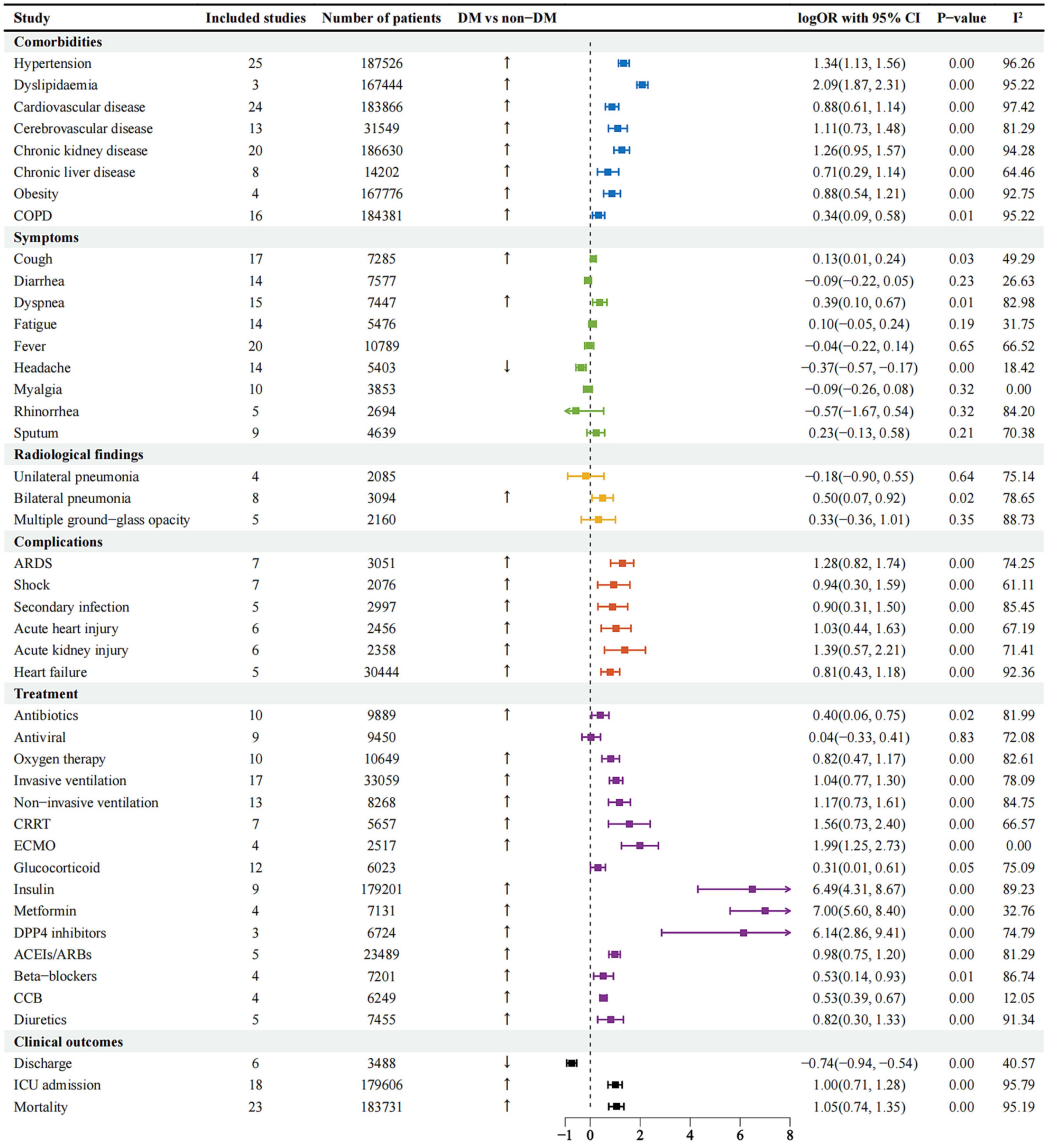
Forest plots comparing comorbidities, symptoms, radiological findings, complications, clinical outcomes and treatment in the DM and non-DM groups of SARS-CoV-2 infected patients. COPD, chronic obstructive pulmonary disease; ARDS, acute respiratory distress syndrome; CRRT, continuous renal replacement therapy; ECMO, extracorporeal membrane oxygenation; DPP4, dipeptidyl peptidase-4; ACEs, angiotensin-converting enzyme; ARBs, angiotensin II receptor blockers; CCB, calcium channel blocker.

After the analysis, as can be seen in Supplementary Table 2, it can be observed that the age and BMI of SARS-CoV-2 infected people in the DM group are older, and the length of hospitalization in this group is longer compared to the non-DM group. On admission, there were no significant differences in body temperature, heart rate, diastolic blood pressure between the DM group and the non-DM group (all *p* > 0.05), but there were obvious differences in respiratory rate and systolic blood pressure (all *p* = 0.00). A higher incidence in men than in women was seen in diabetic patients infected with SARS-CoV-2 [0.46, 95% CI (0.2–0.71%), I^2^-97.81%], with hypertension being the most common comorbidity [1.34, 95% CI (1.13–1.56%), I^2^-96.26%], followed by cerebrovascular disease [1.11, 95%CI (0.73–1.48%), I^2^-81.29%] and CKD [1.26, 95% CI (0.95 ~ 1.57%), I^2^-94.28%]. Interestingly, the incidence of DM combination with COPD was minimal (Figure 2), with dyslipidaemia [2.09, 95% CI (1.87–2.31%), I^2^-95.22%] having the highest probability, but the included studies were few and could be validated by continuing to observe other studies. The most common symptoms were dyspnea [0.39, 95% CI (0.10–0.67%), I^2^-82.98%], cough [0.13, 95% CI (0.01–0.24%), I^2^-49.29%]. An increased incidence of headache [−0.37, 95% CI (−0.57 ~ −0.17%), I^2^-96.26%] was seen in the non-DM group.

### Complications and treatment

Common complications in patients with SARS-CoV-2 infection include ARDS, shock, acute kidney injury, acute heart injury and secondary infection (Figure 2). Patients with DM were more likely to develop ARDS, acute kidney injury and acute cardiac injury, while shock and secondary infection were increased markedly compared with non-DM patients (all, *p* = 0.00).

In terms of treatment, patients in the DM group were more likely to receive antibiotics, antiviral therapy, systemic corticosteroids, high-flow oxygen therapy, mechanical ventilation including invasive and non-invasive ventilation, ECMO, CRRT (Figure 2). After statistical analysis, in terms of hypoglycemic therapy, insulin, metformin and DPP4 inhibitors are the most used in patients. When patients have hypertension, ACEIs/ARBs are the first choice, followed by Beta-blockers, CCB, and Diuretics (Figure 2).

### Radiology and laboratory test results

**Figure 3:**
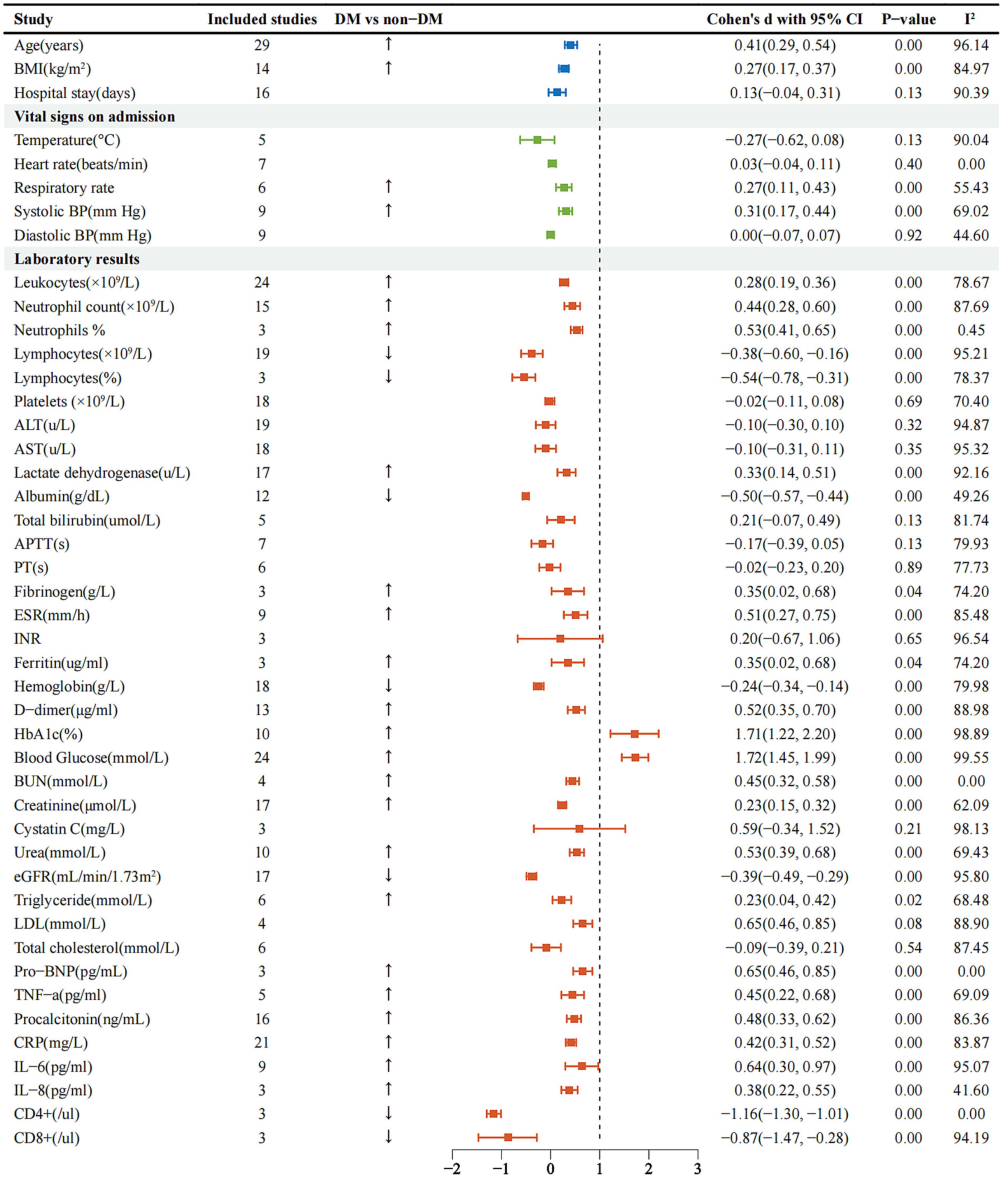
Forest plots comparing admission signs and laboratory tests in the DM and non-DM groups of SARS-CoV-2 infected patients. BMI, body mass index; BP, blood pressure; ALT, alanine aminotransferase; AST, aspartate aminotransferase; APTT, activated partial thromboplastin time; PT, prothrombin time; ESR, erythrocyte sedimentation rate; INR, international normalized ratio; HbA1c, hemoglobin A1c; BUN, blood urea nitrogen; eGFR, estimated glomerular filtration rate; LDL, low-density lipoprotein; BNP, brain natriuretic peptide; TNF, tumor necrosis factor; CRP, C-reactive protein; IL, interleukin.

As we can see in Supplementary Table 1, the most common imaging finding was bilateral pulmonary infiltrates [0.50, 95%CI (0.07–0.92%), I^2^-78.65%]. Regarding the laboratory test results, we could find that the DM group patients had increased white blood cell count, neutrophil count, neutrophil%, fibrinogen, ferritin, D-dimer, ESR and higher levels of Pro-BNP in routine blood tests, however, lymphocyte count, platelets, hemoglobin decreased (Figure 3), lactate dehydrogenase [0.33, 95%CI (0.14 ~ 0.51%), I^2^-92.16%] was significantly increased, albumin [−0.50, 95%CI (−0.57 ~ −0.44%), I^2^-49.26%] was decreased. Compared with non-DM group, creatinine [−0.23, 95%CI (0.15 ~ 0.32%), I^2^-62.09%] was strikingly higher, eGFR [−0.39, 95% CI (−0.49 ~ −0.29%), I^2^-95.80%] showed a decline. The results of blood lipid analysis showed that triglyceride [0.23, 95%CI (0.04 ~ 0.42%), I^2^-68.48%] maintained a peak level. Inflammatory markers such as tumor necrosis factor alpha (TNF-*α*), procalcitonin, C-reactive protein (CRP), interleukin 6 (IL-6) and IL-8 were dramatically improved (Figure 3), but CD4+ and CD8+ were definitely reduced. We subgroup analysis of D-dimer (Supplementary Figure 1), < 1ug/ml was [0.35, 95%CI (−0.18 ~ 0.89%), I2-91.90%], and ≥ 1ug/ml was [0.57, 95% CI (0.38 ~ 0.67%), I2-88.45%]. We then performed a subgroup analysis of ESR (Supplementary Figure 2), < 40 mm/h was [0.44, 95%CI (0.23 ~ 0.65%), I2-66.89%], ≥ 40 mm/h was [0.58, 95%CI (0.37 ~ 0.80%), I2-74.25%], and finally subgroup analysis of hemoglobin A1c (HbA1c) (Supplementary Figure 3), < 7.5% was [1.19, 95%CI (0.42–1.96%), I^2^-96.92%], ≥ 7.5% was [2.05, 95% CI (1.51 ~ 2.58%), I^2^-98.33%].

### Clinical outcome

Outcomes of COVID-19 patients included ICU admission [1.00, 95%CI (0.71–1.28%), I^2^-95.79%], hospital discharge [−0.74, 95% CI (−0.94–0.54%), I^2^-40.75%] and death [1.05, 95%CI (0.74–1.35%), I^2^-95.19%] (Supplementary Table 1). The DM group had lower discharge rates and higher death rates than the non-DM group. At the same time, a large number of diabetic patients were transferred to the ICU for additional treatment.

## Discussion

The rapid global spread of COVID-19 suggests that SARS-CoV-2 has a strong transmission potential in humans. In this systematic review and meta-analysis of 32 studies and 192,693 patients, we systematically reviewed and analyzed numerous clinical and laboratory signatures of predisposition leading to COVID-19 related mortality. Multiple lines of evidence suggest that diabetes is one of the major risks of death in COVID-19 patients, and is considered to be the underlying mechanism of microvascular disease, endothelial dysfunction, severe pneumonia, inflammatory storm, which underlie the adverse outcomes of COVID-19 (12, 13). To the best of our knowledge, this meta-analysis leverages the largest number of studies and largest sample size to date to assess the association between disease severity and mortality risk in COVID-19. Our findings suggest that diabetes in COVID-19 patients is associated with an increased risk of serious infection and mortality compared to non-diabetic patients. Our study provides evidence of how diabetes mediates outcomes in hospitalized adults with COVID-19.

Upon analysis, it was found that the infected patients in the DM group were older, had a higher BMI and were mostly male compared to those without DM, suggesting that they were at higher risk of SARS-CoV-2 infection, with more males than females. This may be due to the fact that females produce extra strong immune response, as estrogen and progesterone can help increase innate and adaptive immune responses, estrogen also promotes B-cell activation, maturation (14, 15). Hypertension is commonly reported as the most common disease associated with COVID-19 patients. It is also an independent risk factor for higher mortality and morbidity in patients with coronavirus infection (16), persistent hyperglycemia and metabolic changes in patients with diabetes and coexisting risk factors. Hypertension causes microvascular changes as well as macrovascular changes, creating a vicious cycle that also leads to cardiovascular events. Therefore, additional attention should be paid to diabetics with underlying comorbidities, especially hypertension. The analysis found that the prevalence of high blood pressure, cerebrovascular disease, chronic kidney disease was significantly higher in the large number of diabetic patients infected with SARS-CoV-2, who were also older than the non-diabetic patients. Diabetes-related comorbidities and uncontrolled hyperglycemia increase the risk of composite endpoints and mortality in COVID-19 patients, especially the increased cardiovascular risk associated with diabetes and hypertension, which additionally contribute to poor outcomes in COVID-19. Common early symptoms of COVID-19 patients include fever, cough, sputum production and additional symptoms of lower respiratory tract infection. As the most common symptom, more than 80% of patients had a fever, but about 40% had a fever on admission, indicating that many patients had intermittent fevers. However, in this meta-analysis there can be no differences in fever between the two groups with cough and dyspnea (Supplementary Table 2). Radiographic findings hinted that bilateral pneumonia obtained on chest CT was more prevalent in diabetic patients, suggesting that these patients had more severe pneumonia.

The COVID-19 virus spreads through the respiratory mucosa and induces a cytokine storm in the body, producing a series of immune responses that alter peripheral white blood cells and lymphocytes, thus increasing inflammation levels. Cell counts increased but lymphocyte counts were significantly lower. The findings may suggest that people with diabetes are more susceptible to viral infections and more susceptible to bacterial infections. Hyperglycemia inhibits neutrophil chemotaxis, reduces phagocytosis of neutrophils, macrophages, monocytes, and impairs cell-mediated immunity (17). The reduction in lymphocyte counts indicates that SARS-CoV-2 depletes immune cells and suppresses the body’s immune function. In addition, severe patients had significantly fewer lymphocytes than non-severe patients, suggesting that the degree of lymphocyte decline can be used to assess the severity of the disease. The continued decline of lymphocytes in the cells is also an indicator of disease progression. The levels of inflammatory markers including CRP, erythrocyte sedimentation rate (ESR), TNF-*α*, Procalcitonin, IL-6, and IL-8 in diabetic patients were significantly higher than those in non-diabetic patients, while CD4+ and CD8+ were lower than in the control group (Supplementary Table 3). CRP is simply an inflammatory biochemical marker, elevated levels of CRP hint the introduction of a cytokine storm by 2019-nCoV, which is critical for the progression of 2019-nCoV. A higher PCT indicates an increased risk of systemic infection and sepsis among diabetic patients infected with COVID-19. Elevated glucose levels directly induce viral replication and pro-inflammatory cytokine expression, which primarily affect lymphocytes, especially T cells, with an increased proportion of pro-inflammatory Th17 CD4+ T cells and cytokine levels. CD4+ and CD8+ peripheral counts of T cells decreased. Meanwhile, viral infection promotes T cell programmed cell death protein 1 (PD-1) expression. Thus, hyperglycemic patients may exhibit impaired antiviral interferon responses and delayed Th1/Th17 activation, which lead to hyperinflammatory responses (18, 19), it may explain why blood glucose levels are elevated during SARS-CoV-2 infection cause T cell dysfunction and lymphopenia. Studies have shown that hyperglycemia plays a deleterious role in the overproduction of IL-6, which is associated with increased lung infiltration and severity of COVID-19, for elevated IL-6, anti-IL-6 therapeutic strategies (Tocilizumab or Janus kinase inhibitors) may be particularly effective in DM patients with severe COVID-19 (12, 20). One study pointed out that inflammatory markers such as CRP levels, serum ferritin and ESR in COVID-19 cases were positively correlated with glycated hemoglobin, while SaO2 was negatively correlated with glycated hemoglobin (21), therefore, low and elevated HbA1c levels may have a positive correlation. Identification of risk of death and adverse outcomes in hospitalized COVID-19 patients. A recent study showed that even patients with diabetes who had properly-controlled HbA1c (6–7%) had a risk of serious infections compared with patients without diabetes, and that this risk increased with increased HbA1c (22). At the same time, hypoglycemia or hyperglycemia is associated with poor prognosis and poor clinical outcomes. Some studies on the management of hospitalized patients with hyperglycemia (especially in the ICU setting) suggest that blood glucose levels should be maintained between 7.8-10 mmol/L to avoid excessive hyperglycemia or moderate/severe hypoglycemia, preventing multiple organ failure and fatal consequences. Second, DM may induce clotting in COVID-19 patients, especially D-dimer produced from fibrin degradation, reflecting the severity of the clotting condition. In addition to deep vein thrombosis, elevated D-dimer can be the expression of capillary microthrombi, which leads to an increased risk of death due to pulmonary capillary endothelial damage (23), some preventive regimens should be taken in clinical work. In addition, indicators of kidney injury, including serum creatinine and blood urea nitrogen are associated with higher mortality in patients with COVID-19, plenty of patients with diabetes have significantly lower eGFR on admission compared with non-diabetic patients, which is due to the incidence of acute kidney injury in patients with diabetes higher than non-diabetic patients. Diabetics commonly develop a chronic inflammatory condition. It makes these patients more vulnerable to the devastating effects of the so-called COVID-19 cytokine storm, causing multiple organ damage and secondary pathophysiological changes in tissues (24), leading to severe complications such as ARDS, shock, acute heart and kidney damage in novel coronavirus pneumonia. Respiratory support for patients with RSV is critical to reducing mortality because the disease is so severe. It is essential to note that most patients require hyperbaric oxygen therapy. Some patients require mechanical ventilation, both invasive and noninvasive. As can be seen from the data, infected patients in the diabetes group required more mechanical ventilation. We found that patients with diabetes were more likely to be transferred to the ICU and were treated most frequently with antibiotics, antivirals, corticosteroids, and especially advanced life support including ECMO, mechanical ventilation, and continuous renal replacement therapy. Intravenous corticosteroids are indicated primarily for acute respiratory distress syndrome in mechanically ventilated patients with novel coronavirus pneumonia. They are administered in the shortest amount of time to reduce side effects. In terms of hypoglycemia treatment, current recommendations for hypoglycemia medication for diabetics with COVID-19 mainly contain the use of metformin and DPP-4 inhibitors for mild cases and the addition of insulin for severe cases. In terms of antihypertensive therapy, angiotensin converting enzyme 2 (ACE2) may genuinely protect against severe respiratory infections by converting angiotensin II to angiotensin with significant anti-inflammatory properties, so an angiotensin converting enzyme inhibitors (ACEI) that results in increased ACE2 expression may really be beneficial, using ACEI or angiotensin II receptor blockers (ARBs) may benefit COVID-19 outcomes and positively modulate its outcomes, the recent meta-analyses further support the role of ACEIs and ARBs in disease progression beneficial effect (25). Studies have suggested that another significant factor contributing to poor outcomes is the use of beta-blockers in hospitalized COVID-19 patients, although controversial, *β*-blockers may be beneficial because they reduce pulmonary vascular flow, ultimately reducing additional damage to the lungs of patients with suspected ARDS (26).

In summary, this is the largest meta-analysis to date of the clinical characteristics and outcomes of diabetic patients infected with SARS-CoV-2. A large global multicenter study of data showed that patients with diabetes who were hospitalized with COVID-19 had an increased risk of death, lower hospital discharge rates and higher ICU admission rates than patients without diabetes. Hypertension, cerebrovascular disease, CKD, higher levels of inflammatory markers, and multiple complications in COVID-19 patients with diabetes are all predictors of poor outcomes in people with diabetes. Our findings will help identify elevated risk factors in diabetes patients, which will aid in early prediction, accurate diagnosis and treatment of COVID-19 patients.

### Limitations and future directions

There are several limitations to our study. First, we find significant heterogeneity between studies and significant publication bias in several variables. This may be explained by differences in study design, patient population, and sample size. Second, a stratified analysis by type of diabetes is not feasible. Third, although we manually excluded some studies to avoid including any duplicates, it is still possible that some overlapping patients were included in our meta-analysis, which may have slightly affected our results. Fourth, different follow-up periods and missing follow-up information may have skewed some of the results, particularly mortality. Finally, most of the studies included in our meta-analysis were retrospective, but only one was prospective, meaning that the criteria for inclusion in the diabetes group relied primarily on prior clinical history, which would have led us to exclude some cases of original diabetes.

## Data Availability

The original contributions presented in the study are included in the article/Supplementary material, further inquiries can be directed to the corresponding author.

## Supplementary material

SUPPLEMENTARY FIGURE 1 D-dimer was divided into two subgroups of < 1ug/ml and ≥ 1ug/ml, and the figure show the forest plot of the two subgroups analyzed.

SUPPLEMENTARY FIGURE 2 ESR was divided into two subgroups of < 40mm/h and ≥ 40mm/h, and the figure shows the forest plot of the two subgroups analyzed.

SUPPLEMENTARY FIGURE 3 Hemoglobin A1c was divided into two subgroups of < 7.5% and ≥ 7.5%, and the figure shows the forest plot of the two subgroups analyzed.
