# Supplementary figures and images for: The clinical features and outcomes of diabetes patients infected with COVID-19: a systematic review and meta-analysis comprising 192,693 patients
Source: Front Med (Lausanne). 2025 Jan 29;12:1523139. doi: 10.3389/fmed.2025.1523139 (PMC11813781; doi:10.3389/fmed.2025.1523139)

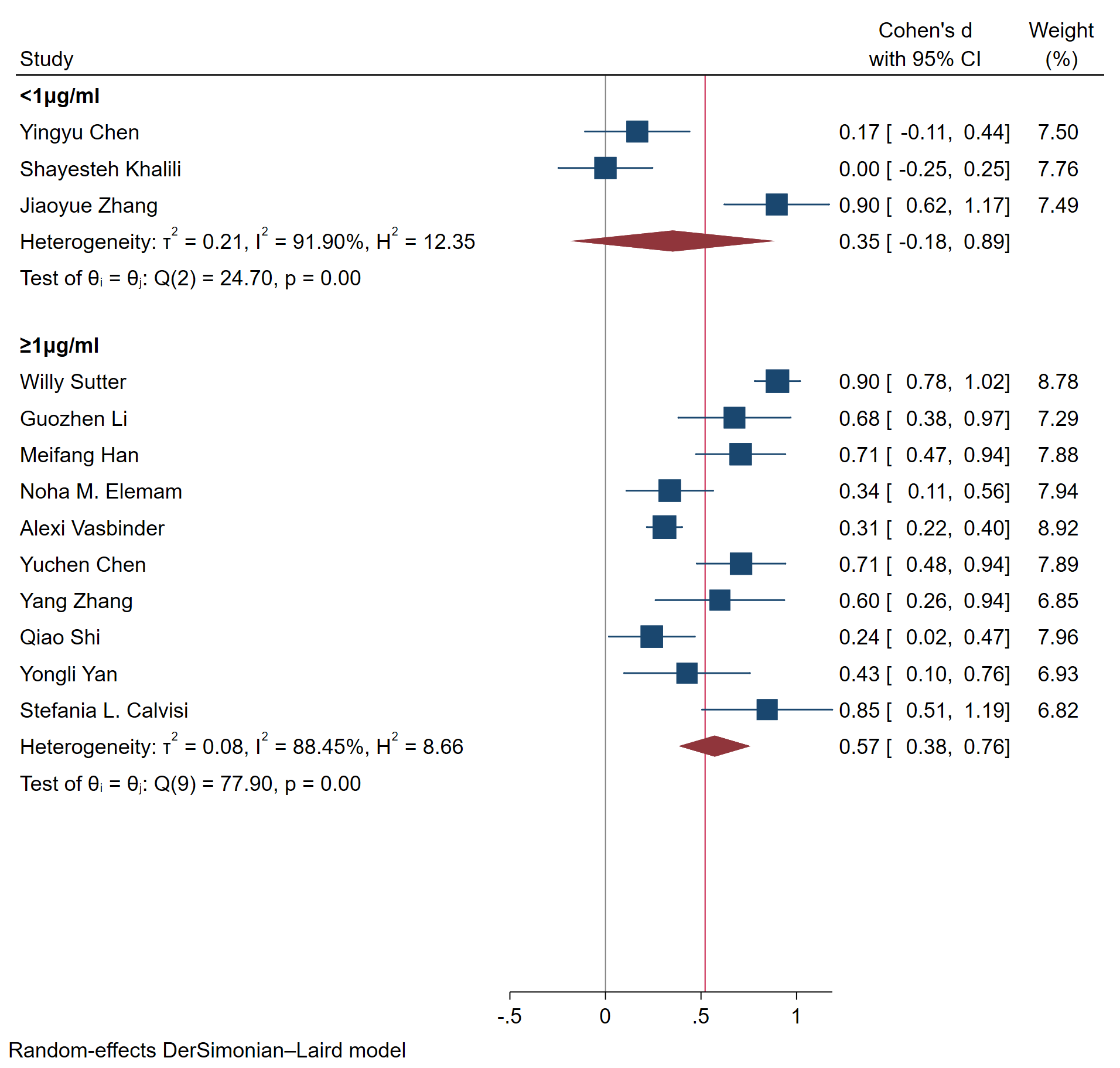

Supplement: SUPPLEMENTARY FIGURE 1 — D-dimer was divided into two subgroups of < 1ug/ml and ≥ 1ug/ml, and the figure show the forest plot of the two subgroups analyzed. [file Image_1.tif]

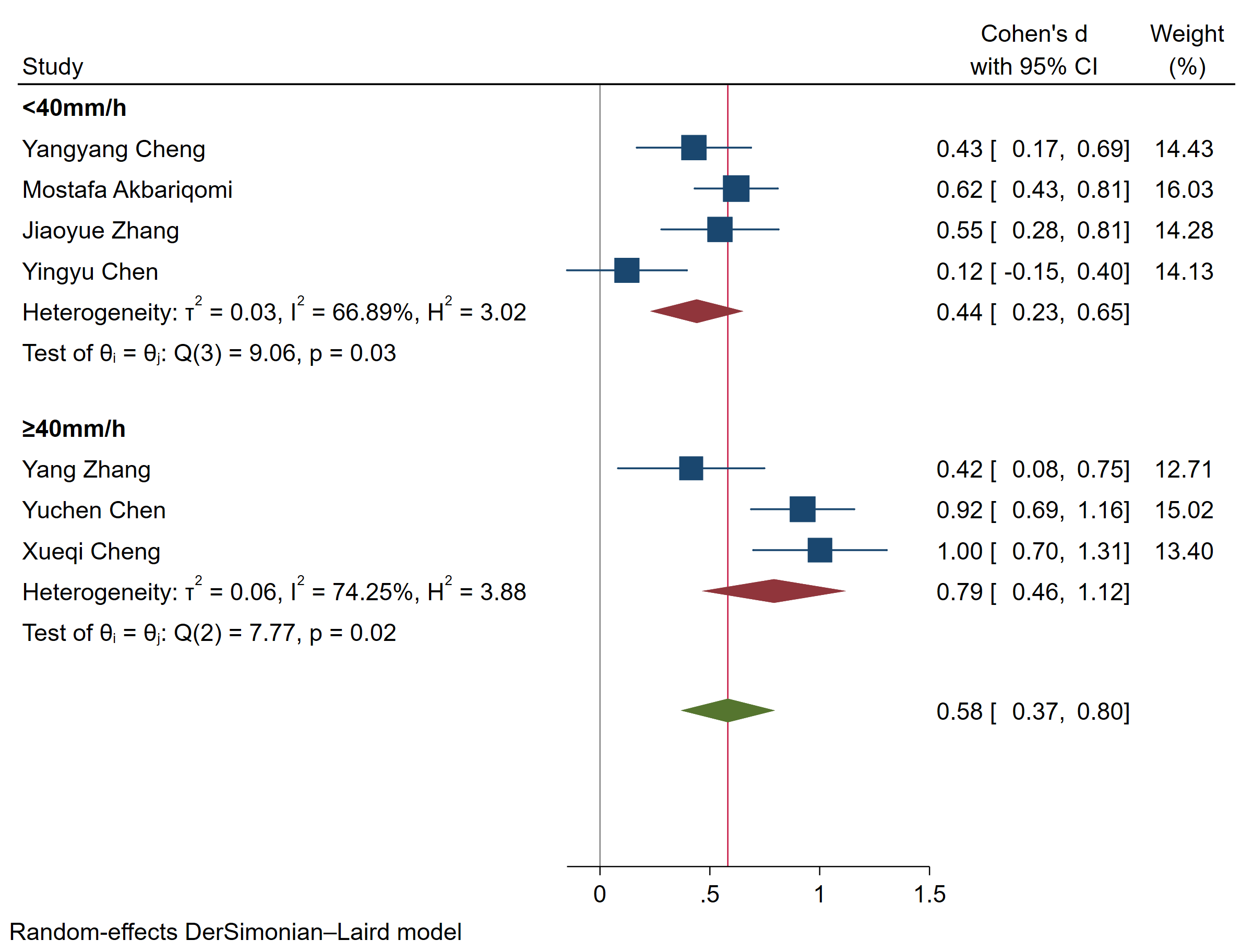

Supplement: SUPPLEMENTARY FIGURE 2 — ESR was divided into two subgroups of < 40mm/h and ≥ 40mm/h, and the figure shows the forest plot of the two subgroups analyzed. [file Image_2.tif]

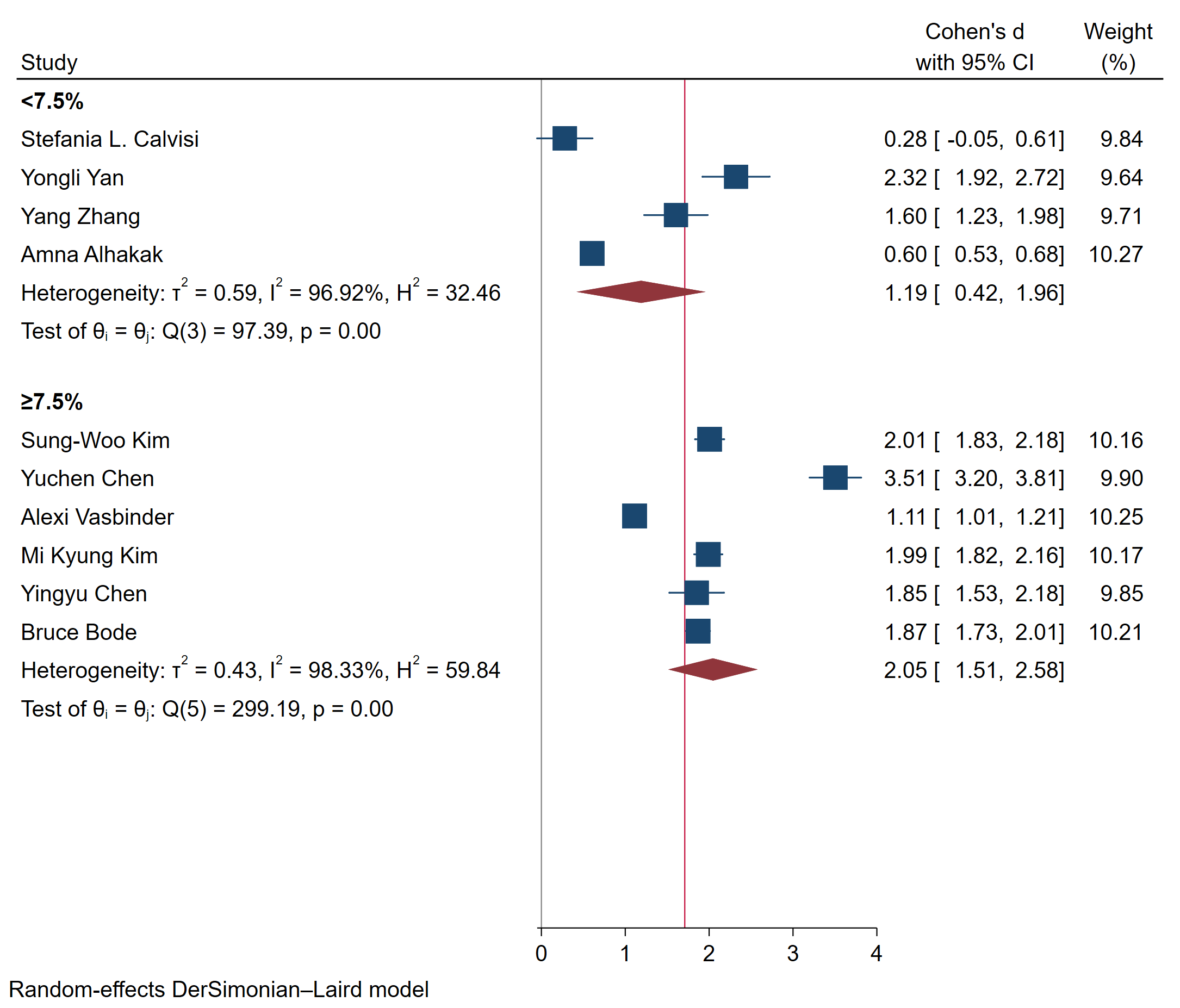

Supplement: SUPPLEMENTARY FIGURE 3 — Hemoglobin A1c was divided into two subgroups of < 7.5% and ≥ 7.5%, and the figure shows the forest plot of the two subgroups analyzed. [file Image_3.tif]
