# Supplementary material for: The clinical features and outcomes of diabetes patients infected with COVID-19: a systematic review and meta-analysis comprising 192,693 patients
Source: Front Med (Lausanne). 2025 Jan 29;12:1523139. doi: 10.3389/fmed.2025.1523139 (PMC11813781; doi:10.3389/fmed.2025.1523139)
Supplement: Supplementary file 4 [file Supplementary_file_1.docx]

| **Clinical characteristics**  **Supplement 1** The difference between DM and non-DM patients in clinical characteristics | **Included**  **studies** | **Heterogeneity**^*^  **(%)** | **logOR** | **95% CI** | **P value** | **Study design** |
| --- | --- | --- | --- | --- | --- | --- |
| Male vs Female(DM group) | 30 | 97.81 | 0.46 | 0.20~0.71 | 0.00 | PS & RS |
| **Comorbidities** |  |  |  |  |  |  |
| Hypertension | 25 | 96.26 | 1.34 | 1.13~1.56 | 0.00 | PS & RS |
| Dyslipidaemia | 3 | 95.22 | 2.09 | 1.87~2.31 | 0.00 | RS |
| Cardiovascular disease | 24 | 97.42 | 0.88 | 0.61~1.14 | 0.00 | PS & RS |
| Cerebrovascular disease | 13 | 81.29 | 1.11 | 0.73~1.48 | 0.00 | RS |
| Chronic kidney disease | 20 | 94.28 | 1.26 | 0.95~1.57 | 0.00 | PS & RS |
| Chronic liver disease | 8 | 64.46 | 0.71 | 0.29~1.14 | 0.00 | PS & RS |
| Heart failure | 5 | 92.36 | 0.81 | 0.43~1.18 | 0.00 | RS |
| Obesity | 4 | 92.75 | 0.88 | 0.54~1.21 | 0.00 | PS & RS |
| COPD | 16 | 95.22 | 0.34 | 0.09~0.58 | 0.01 | PS & RS |
| **Symptoms** |  |  |  |  |  |  |
| Cough | 17 | 49.29 | 0.13 | 0.01~0.24 | 0.03 | PS & RS |
| Diarrhea | 14 | 26.63 | -0.09 | -0.22~0.05 | 0.23 | PS & RS |
| Dyspnea | 15 | 82.98 | 0.39 | 0.10-0.67 | 0.01 | RS |
| Fatigue | 14 | 31.75 | 0.10 | -0.05~0.24 | 0.19 | RS |
| Fever | 20 | 66.52 | -0.04 | -0.22~0.14 | 0.65 | PS & RS |
| Headache | 14 | 18.42 | -0.37 | -0.57~-0.17 | 0.00 | RS |
| Myalgia | 10 | 0.00 | -0.09 | -0.26~0.08 | 0.32 | PS & RS |
| Rhinorrhea | 5 | 84.2 | -0.57 | -1.67~0.54 | 0.32 | RS |
| Sputum | 9 | 70.38 | 0.23 | -0.13~0.58 | 0.21 | PS & RS |
| **Radiological findings** |  |  |  |  |  |  |
| Unilateral pneumonia | 4 | 75.14 | -0.18 | -0.90~0.55 | 0.64 | PS & RS |
| Bilateral pneumonia | 8 | 78.65 | 0.5 | 0.07~0.92 | 0.02 | PS & RS |
| Multiple ground-glass opacity | 5 | 88.73 | 0.33 | -0.36~1.01 | 0.35 | RS |
| **Complications** |  |  |  |  |  |  |
| ARDS | 7 | 74.25 | 1.28 | 0.82~1.74 | 0.00 | RS |
| Shock | 7 | 61.11 | 0.94 | 0.30~1.59 | 0.00 | RS |
| Secondary infection | 5 | 85.45 | 0.90 | 0.31~1.50 | 0.00 | RS |
| Acute heart injury | 6 | 67.19 | 1.03 | 0.44~1.63 | 0.00 | RS |
| Acute kidney injury | 6 | 71.41 | 1.39 | 0.57~2.21 | 0.00 | RS |
| **Treatment** |  |  |  |  |  |  |
| Antibiotics | 10 | 81.99 | 0.40 | 0.06~0.75 | 0.02 | PS & RS |
| Antiviral | 9 | 72.08 | 0.04 | -0.33~0.41 | 0.83 | PS & RS |
| Oxygen therapy | 10 | 82.61 | 0.82 | 0.47~1.17 | 0.00 | PS & RS |
| Invasive ventilation | 17 | 78.09 | 1.04 | 0.77~1.30 | 0.00 | PS & RS |
| Non-invasive ventilation | 13 | 84.75 | 1.17 | 0.73~1.61 | 0.00 | PS & RS |
| CRRT | 7 | 66.57 | 1.56 | 0.73~2.40 | 0.00 | RS |
| ECMO | 4 | 0.00 | 1.99 | 1.25~2.73 | 0.00 | RS |
| Glucocorticoid | 12 | 75.09 | 0.31 | 0.01~0.61 | 0.05 | PS & RS |
| Insulin | 9 | 89.23 | 6.49 | 4.31~8.67 | 0.00 | PS & RS |
| Metformin | 4 | 32.76 | 7.00 | 5.60~8.40 | 0.00 | PS & RS |
| DPP4 inhibitors | 3 | 74.79 | 6.14 | 2.86~9.41 | 0.00 | PS & RS |
| ACEIs/ARBs | 5 | 81.29 | 0.98 | 0.75~1.2 | 0.00 | RS |
| Beta-blockers | 4 | 86.74 | 0.53 | 0.14~0.93 | 0.01 | RS |
| CCB | 4 | 12.05 | 0.53 | 0.39~0.67 | 0.00 | RS |
| Diuretics | 5 | 91.34 | 0.82 | 0.30~1.33 | 0.00 | RS |
| **Clinical outcomes** |  |  |  |  |  |  |
| Discharge | 6 | 40.57 | -0.74 | -0.94~-0.54 | 0.00 | PS & RS |
| ICU admission | 18 | 95.79 | 1.00 | 0.71~1.28 | 0.00 | PS & RS |
| Mortality | 23 | 95.19 | 1.05 | 0.74~1.35 | 0.00 | PS & RS |

*, heterogeneity: I2 <50%, P>0.1, suggesting that the homogeneity of each test was good, and the meta-analysis was performed using the fixed

Effect model (FE), while in contrast (I2 >50%, P<0.1), the random effects model (RE) was used. P<0.05 was considered statistically significant.

COPD, chronic obstructive pulmonary disease;ARDS, acute respiratory distress syndrome; CRRT, continuous renal replacement therapy; ECMO, extracorporeal membrane oxygenation; ICU admission,intensive care unit admission; DPP-4, dipeptidyl peptidase-4; ACEI/ARB: angiotensin

converting enzyme inhibitor/angiotensin II receptor blocker; CCB: calcium channel blocker; NA, not available. PS: Prospective Studies; RS:

Retrospective Studies.
