# Supplementary material for: The clinical features and outcomes of diabetes patients infected with COVID-19: a systematic review and meta-analysis comprising 192,693 patients
Source: Front Med (Lausanne). 2025 Jan 29;12:1523139. doi: 10.3389/fmed.2025.1523139 (PMC11813781; doi:10.3389/fmed.2025.1523139)
Supplement: Supplementary file 5 [file Supplementary_file_2.docx]

| **Clinical characteristics**  **Supplement 2** The difference between DM and non-DM patients in vital signs and Laboratory results | **Included**  **studies** | **Heterogeneity**^*^  **(%)** | **SMD** | **95% CI** | **P value** | **Egger's test P value** |
| --- | --- | --- | --- | --- | --- | --- |
| BMI(kg/m^2^) | 14 | 84.97 | 0.27 | 0.17~0.37 | 0.00 | 0.1472 |
| Age(years) | 29 | 96.14 | 0.41 | 0.29~0.54 | 0.00 | 0.6936 |
| Hospital stay(days) | 16 | 90.39 | 0.13 | -0.04~0.31 | 0.13 | 0.3664 |
| **Vital signs on admission** |  |  |  |  |  |  |
| Body temperature(°C) | 5 | 90.04 | -0.27 | -0.62~0.08 | 0.13 | NA |
| Heart rate(beats/min) | 7 | 0.00 | 0.03 | -0.04~0.11 | 0.40 | NA |
| Respiratory rate | 6 | 55.43 | 0.27 | 0.11~0.43 | 0.00 | NA |
| Systolic BP(mm Hg) | 9 | 69.02 | 0.31 | 0.17~0.44 | 0.00 | NA |
| Diastolic BP(mm Hg) | 9 | 44.60 | 0.00 | -0.07~0.07 | 0.92 | NA |
| **Laboratory results** |  |  |  |  |  |  |
| Leukocytes (×10^9^/L) | 24 | 78.67 | 0.28 | 0.19~0.36 | 0.00 | 0.0249 |
| Neutrophil count(×10^9^/L) | 15 | 87.69 | 0.44 | 0.28~0.60 | 0.00 | 0.0582 |
| Neutrophils (%) | 3 | 0.45 | 0.53 | 0.41~0.65 | 0.00 | NA |
| Lymphocytes (×10^9^/L) | 19 | 95.21 | -0.38 | -0.60~-0.16 | 0.00 | 0.1571 |
| Lymphocytes(%) | 3 | 78.37 | -0.54 | -0.78~-0.31 | 0.00 | NA |
| Platelets (×10^9^/L) | 18 | 70.40 | -0.02 | -0.11~0.08 | 0.69 | 0.1894 |
| ALT(u/L) | 19 | 94.87 | -0.10 | -0.30~0.10 | 0.32 | 0.7938 |
| AST(u/L) | 18 | 95.32 | -0.10 | -0.31~0.11 | 0.35 | 0.8757 |
| Lactate dehydrogenase(u/L) | 17 | 92.16 | 0.33 | 0.14~0.51 | 0.00 | 0.3635 |
| Albumin(g/dL) | 12 | 49.26 | -0.50 | -0.57~-0.44 | 0.00 | 0.0056 |
| Total bilirubin(umol/L) | 5 | 81.74 | 0.21 | -0.07~0.49 | 0.13 | NA |
| APTT(s) | 7 | 79.93 | -0.17 | -0.39~0.05 | 0.13 | NA |
| PT(s) | 6 | 77.73 | -0.02 | -0.23~0.20 | 0.89 | NA |
| Fibrinogen(g/L) | 3 | 74.2 | 0.35 | 0.02~0.68 | 0.04 | NA |
| ESR(mm/h) | 9 | 85.48 | 0.51 | 0.27~0.75 | 0.00 | NA |
| INR | 3 | 96.54 | 0.20 | -0.67~1.06 | 0.65 | NA |
| Ferritin(ug/ml) | 3 | 74.20 | 0.35 | 0.02~0.68 | 0.04 | NA |
| Hemoglobin(g/L) | 18 | 79.98 | -0.24 | -0.34~-0.14 | 0.00 | 0.2081 |
| D-dimer(μg/ml) | 13 | 88.98 | 0.52 | 0.35~0.70 | 0.00 | 0.8112 |
| HbA1c(%) | 10 | 98.89 | 1.71 | 1.22~2.20 | 0.00 | NA |
| Blood glucose(mmol/L) | 24 | 99.55 | 1.72 | 1.45~1.99 | 0.00 | 0.0000 |
| BUN(mmol/L) | 4 | 0.00 | 0.45 | 0.32~0.58 | 0.00 | NA |
| Creatinine(μmol/L) | 17 | 62.09 | 0.23 | 0.15~0.32 | 0.00 | 0.5690 |
| Cystatin C(mg/L) | 3 | 98.13 | 0.59 | -0.34~1.52 | 0.21 | NA |
| Urea(mmol/L) | 10 | 69.43 | 0.53 | 0.39~0.68 | 0.00 | NA |
| eGFR(mL/min/1.73m^2^) | 17 | 95.80 | -0.39 | -0.49~-0.29 | 0.00 | 0.4819 |
| Triglyceride(mmol/L) | 6 | 68.48 | 0.23 | 0.04~0.42 | 0.02 | NA |
| LDL(mmol/L) | 4 | 88.90 | 0.33 | -0.04~0.71 | 0.08 | NA |
| Total cholesterol(mmol/L) | 6 | 87.45 | -0.09 | -0.39~0.21 | 0.54 | NA |
| Pro-BNP(pg/mL) | 3 | 0.00 | 0.65 | 0.46~0.85 | 0.00 | NA |
| TNF-α(pg/ml) | 5 | 69.09 | 0.45 | 0.22~0.68 | 0.00 | NA |
| Procalcitonin(ng/mL) | 16 | 86.36 | 0.48 | 0.33~0.62 | 0.00 | 0.8639 |
| CRP(mg/L) | 21 | 83.87 | 0.42 | 0.31-0.52 | 0.00 | 0.0014 |
| IL-6(pg/ml) | 9 | 95.07 | 0.64 | 0.30~0.97 | 0.00 | NA |
| IL-8(pg/ml) | 3 | 41.60 | 0.38 | 0.22~0.55 | 0.00 | NA |
| CD4+(/ul) | 3 | 0.00 | -1.16 | -1.30~-1.01 | 0.00 | NA |
| CD8+(/ul) | 3 | 94.19 | -0.87 | -1.47~-0.28 | 0.00 | NA |

*, heterogeneity: I2 <50%, P>0.1, suggesting that the homogeneity of each test was good, and the meta-analysis was performed using the fixed

Effect model (FE), while in contrast (I2 >50%, P<0.1), the random effects model (RE) was used. P<0.05 was considered statistically significant.

AST, aspartate aminotransferase; ALT, alanine aminotransaminase; HbA1c, glycosylated hemoglobin; eGFR, estimated glomerular filtration rate;

APTT, activated partial thromboplastin time; PT, prothrombin time; INR,international normalized ratio; ESR, erythrocyte sedimentation rate;

CRP, C-reactive protein; BUN, blood urea nitrogen; pro-BNP, precursor of the brain natriuretic peptide; LDL: low-density lipoprotein; TNF: tumor

necrosis factor; IL: interleukin; Blood glucose: includes fasting blood glucose and random blood glucose.

.
