# Supplementary material for: The clinical features and outcomes of diabetes patients infected with COVID-19: a systematic review and meta-analysis comprising 192,693 patients
Source: Front Med (Lausanne). 2025 Jan 29;12:1523139. doi: 10.3389/fmed.2025.1523139 (PMC11813781; doi:10.3389/fmed.2025.1523139)
Supplement: Supplementary file 6 [file Supplementary_file_3.docx]

**Supplement 3** Systematic evaluation of all studies by NOS tool

| Study | Selection | | | | Comparability | Exposure | | |
| --- | --- | --- | --- | --- | --- | --- | --- | --- |
|  | Is the case definition adequate? | Representativeness of the cases | Selection of Controls | Definition of Controls | Comparability of cases and controls on the basis of the design or analysis | Ascertainment of exposure | Same method of ascertainment for cases and controls | Non-Response rate |
| Calvisi et al.,2021 | ☆ | ☆ | ☆ |  | ☆ ☆ | ☆ |  | ☆ |
| Cheng et al.,2020 | ☆ | ☆ | ☆ | ☆ | ☆ |  | ☆ | ☆ |
| Yan et al.,2020 | ☆ | ☆ | ☆ | ☆ | ☆ ☆ | ☆ |  | ☆ |
| Shi et al.,2020 | ☆ | ☆ | ☆ | ☆ | ☆ ☆ | ☆ | ☆ | ☆ |
| Zhang et al.,2020 | ☆ | ☆ | ☆ | ☆ | ☆ ☆ | ☆ | ☆ | ☆ |
| Kim SW et al.,2021 | ☆ | ☆ | ☆ |  | ☆ | ☆ | ☆ | ☆ |
| Akbariqomi et al.,2020 | ☆ | ☆ | ☆ | ☆ | ☆ ☆ | ☆ | ☆ | ☆ |
| Cai et al.,2020 | ☆ | ☆ | ☆ |  | ☆ ☆ | ☆ | ☆ | ☆ |
| Chen et al.,2020 | ☆ | ☆ | ☆ | ☆ | ☆ ☆ | ☆ | ☆ | ☆ |
| Vasbinder et al.,2022 | ☆ | ☆ | ☆ | ☆ | ☆ ☆ |  |  | ☆ |
| Elemam et al.,2021 | ☆ | ☆ | ☆ | ☆ | ☆ ☆ |  | ☆ | ☆ |
| Cheng et al.,2021 | ☆ | ☆ | ☆ |  | ☆ ☆ | ☆ | ☆ | ☆ |
| Zhang et al.,2020 | ☆ | ☆ | ☆ |  | ☆ ☆ | ☆ | ☆ | ☆ |
| Ling et al.,2020 | ☆ | ☆ | ☆ | ☆ | ☆ ☆ |  | ☆ |  |
| Kim MK et al.,2020 | ☆ | ☆ | ☆ | ☆ | ☆ ☆ | ☆ |  | ☆ |
| Han et al.,2020 | ☆ | ☆ | ☆ | ☆ | ☆ | ☆ |  | ☆ |
| Li et al.,2020 | ☆ | ☆ | ☆ | ☆ | ☆ | ☆ | ☆ | ☆ |
| You et al.,2020 | ☆ | ☆ | ☆ | ☆ | ☆ ☆ | ☆ | ☆ | ☆ |
| Sun et al.,2020 | ☆ | ☆ | ☆ |  | ☆ | ☆ | ☆ | ☆ |
| Khalili et al.,2020 | ☆ | ☆ | ☆ |  | ☆ ☆ | ☆ | ☆ | ☆ |
| Yang et al.,2021 | ☆ | ☆ | ☆ |  | ☆ | ☆ | ☆ | ☆ |
| Chen et al.,2020 | ☆ | ☆ | ☆ | ☆ | ☆ ☆ | ☆ | ☆ | ☆ |
| Sutter et al.,2021 | ☆ | ☆ | ☆ | ☆ | ☆ ☆ |  | ☆ | ☆ |
| Alguwaihes et al.,2020 | ☆ | ☆ | ☆ | ☆ | ☆ | ☆ | ☆ |  |
| Bode et al.,2020 | ☆ | ☆ | ☆ |  | ☆ | ☆ | ☆ | ☆ |
| Al-Salameh et al.,2021 | ☆ | ☆ | ☆ | ☆ | ☆ ☆ |  | ☆ | ☆ |
| Mansour et al.,2020 | ☆ | ☆ | ☆ | ☆ | ☆ ☆ |  | ☆ | ☆ |
| Chung et al.,2020 | ☆ | ☆ | ☆ | ☆ | ☆ ☆ | ☆ | ☆ | ☆ |
| Alshukry et al.,2021 | ☆ | ☆ | ☆ | ☆ | ☆ |  | ☆ | ☆ |
| Alhakak et al.,2022 | ☆ | ☆ | ☆ |  | ☆ ☆ | ☆ | ☆ | ☆ |
| Sonmez et al.,2021 | ☆ | ☆ | ☆ | ☆ | ☆ ☆ | ☆ | ☆ | ☆ |
| Demirci et al.,2021 | ☆ | ☆ | ☆ |  | ☆ ☆ | ☆ | ☆ | ☆ |
