# Supplementary material for: The clinical features and outcomes of diabetes patients infected with COVID-19: a systematic review and meta-analysis comprising 192,693 patients
Source: Front Med (Lausanne). 2025 Jan 29;12:1523139. doi: 10.3389/fmed.2025.1523139 (PMC11813781; doi:10.3389/fmed.2025.1523139)
Supplement: Supplementary file 8 [file Supplementary_file_5.docx]

MOOSE Statement - Reporting Checklist for Authors, Editors, and Reviewers of Meta-analyses of Observational Studies.

| **Reporting Criteria** | **Reported on Page** |
| --- | --- |
| **Reporting of Background** |  |
| Problem definition | 2 |
| Hypothesis statement | -- |
| Description of Study Outcome(s) | 2 |
| Type of exposure or intervention used | -- |
| Type of study design used | 4 |
| Study population | 4 |
| **Reporting of Search Strategy** |  |
| Qualifications of searchers (eg, librarians and investigators) | 2 |
| Search strategy, including time period included in the synthesis and  keywords | 2 |
| Effort to include all available studies, including contact with authors | 4-5 |
| Databases and registries searched | 2-3 |
| Search software used, name and version, including special features used (eg, explosion) | 2-3 |
| Use of hand searching (eg, reference lists of obtained articles) | 2 |
| List of citations located and those excluded, including justification | Figure 1 |
| Method for addressing articles published in languages other than English | -- |
| Method of handling abstracts and unpublished studies | 2 |
| Description of any contact with authors | -- |
| **Reporting of Methods** |  |
| Description of relevance or appropriateness of studies assembled for assessing the hypothesis to be tested | 2-3 |
| Rationale for the selection and coding of data (eg, sound clinical principles or convenience) | 2-3 |
| Documentation of how data were classified and coded (eg, multiple raters, blinding, and interrater reliability) | 2-3 |
| Assessment of confounding (eg, comparability of cases and controls in studies where appropriate | 2, Table1 |
| Assessment of study quality, including blinding of quality assessors;  stratification or regression on possible predictors of study results | 2-3 |
| Assessment of heterogeneity | 2-3 |
| Description of statistical methods (eg, complete description of fixed or random effects models, justification of whether the chosen models account for predictors of study results, dose-response models, or cumulative meta- analysis) in sufficient detail to be replicated | 2-3 |
| Provision of appropriate tables and graphics | Suppleme-ntal info |
| **Reporting of Results** |  |
| Table giving descriptive information for each study included | Table 1 |
| Results of sensitivity testing (eg, subgroup analysis) | 5-6 |
| Indication of statistical uncertainty of findings | Suppleme-ntal info |
| **Reporting of Discussion** |  |
| Quantitative assessment of bias (eg, publication bias) | 2-3 |
| Justification for exclusion (eg, exclusion of non–English-language  citations) | 2 |
| Assessment of quality of included studies | Suppleme-ntal info |
| **Reporting of Conclusions** |  |
| Consideration of alternative explanations for observed results | 7-9 |
| Generalization of the conclusions (ie, appropriate for the data presented and within the domain of the literature review) | 7-9 |
| Guidelines for future research | 7-9 |
| Disclosure of funding source | 9 |
